# Corneal Artificial Endothelial Layer (EndoArt): Literature Review and Our Experience

**DOI:** 10.3390/jcm13216520

**Published:** 2024-10-30

**Authors:** Davide Romano, Mariacarmela Ventura, Sabrina Vaccaro, Eliana Forbice, Scott Hau, Francesco Semeraro, Vito Romano

**Affiliations:** 1 Eye Unit, Department of Medical and Surgical Specialties, Radiological Sciences and Public Health, University of Brescia, 25121 Brescia, Italy; 2 NIHR Biomedical Research Centre, Moorfields Eye Hospital NHS Foundation Trust, UCL Institute of Ophthalmology, London EC1V 9EL, UK; 3 Glaucoma Service, Moorfields Eye Hospital NHS Foundation Trust, London EC1V 2PD, UK; 4 Optometry Education, Moorfields Eye Hospital NHS Foundation Trust, London EC1V 2PD, UK

**Keywords:** chronic corneal oedema, endothelial keratoplasty, artificial endothelial layer, morphological corneal changes, IVCM

## Abstract

**Background/Objectives**: The objective of this study was to examine the morphological corneal changes and outcomes following the implantation of an artificial endothelial layer (EndoArt) in patients with chronic corneal oedema. **Methods**: A systematic review of the literature was conducted alongside a detailed analysis of two clinical cases with chronic corneal oedema that were treated using EndoArt. Our experience with these two cases is included to provide practical insights and real-world outcomes. **Results**: Across the 24 cases reported (including the two presented here), an analysis was possible in 23 cases. Notably, 82% of patients had undergone at least one previous corneal transplant, with 39% having undergone three or more transplants. Additionally, 78% of cases had ocular comorbidities, with glaucoma surgery being the most prevalent (83%), which could have impacted visual outcomes. The follow-up period ranged from 3 to 17 months with a median of 3 months. After EndoArt implantation, the average reduction in the central corneal thickness (CCT) was 29%, and the rebubbling rate was 47.8%, with some cases requiring no rebubbling, while others required it up to 100% of the time. Visual acuity significantly improved from a mean best-corrected visual acuity (BCVA) value of 1.61 ± 0.5 logMAR to 1.07 ± 0.59 logMAR (*p* < 0.001). The CCT decreased from 771 ± 146 µm to 580 ± 134 µm (*p* < 0.001). These findings are consistent with our experience. **Conclusions**: EndoArt shows promise as an alternative treatment for chronic corneal oedema in complex cases where conventional corneal transplantation has failed or carries a high risk of failure. The morphological changes observed using anterior segment optical coherence tomography (OCT) and in vivo confocal microscopy (IVCM) were similar to those reported after endothelial keratoplasty, with the notable exception of the absence of the hyper-reflective donor–host interface.

## 1. Introduction

The gradual endothelial cell loss of transplanted cornea, with progressive stromal and epithelial oedema and loss of corneal clarity, is the main cause of late graft failure. This results in the requirement for re-grafting with a higher risk of unsuccessful subsequent transplant [1,2].

EndoArt (EyeYon Medical, Ness Ziona, Israel) is a new artificial endothelial layer device, and its efficacy in the management of chronic corneal oedema is starting to be reported [3,4,5,6,7,8,9]. The device, made of a copolymer of hydroxyethyl methacrylate and methyl methacrylate, has a dome-shaped profile with a diameter of 6.50 mm and a thickness of 50 μm. It is designed to match the posterior curvature of the cornea and serves as an artificial fluid barrier within the recipient’s posterior stroma, effectively replacing the diseased endothelium. EndoArt functions by decreasing aqueous influx into the central stroma, thereby helping to diminish oedema and restore corneal homeostasis [3].

There is, however, still a lack of reports on the comparative corneal morphological changes evaluated in in vivo confocal microscopy (IVCM) and anterior segment optical coherence tomography (AS-OCT) before and after EndoArt implantation. In view of this, we present a case series showing a comparison of the morphological changes in two patients who underwent EndoArt implantation after chronic endothelial corneal oedema followed by multiple failed transplants and a review of the current literature on EndoArt.

## 2. Materials and Methods

A literature review was performed by a single investigator (DR) using the MEDLINE database (via PubMed) to search for and identify articles for inclusion in this review. Keywords used were “EndoArt” OR “artificial endothelial implant”. Articles, including case series and case reports, up to October 2024, were included.

## 3. Results

### 3.1. Case Series

A 75-year-old female (Case 1) and a 67-year-old male (Case 2) were referred to our clinic in April 2024 and May 2024 with a 1-year history of progressive chronic corneal oedema in the left eye (LE) secondary to failed penetrating keratoplasty (PK) after cataract surgery (Case 1) and failed Descemet stripping automated endothelial keratoplasty (DSAEK) (Case 2). Both of their past ocular history was positive for multiple corneal transplants in the LE.

In Case 1, two DSAEK procedures for endothelial corneal decompensation were performed in 2009 and 2015, followed by 8.25 mm PK for corneal perforation due to infectious keratitis in 2018. Cataract surgery was performed in May 2020; following this, the patient likely developed progressive graft failure. Her history in the right eye (RE) was unremarkable.

In Case 2, three 8.50 mm PKs were performed. The first in 2004 for herpetic keratitis, followed by a second and a third PK for post-graft rejection failure in 2017 and 2020. DSAEK was performed in April 2023 for the management of corneal oedema caused by the third PK failure.

Upon examination, the best-corrected visual acuity (BCVA) in the affected eyes was 1.50 logMAR in Case 1 counting fingers (CF) at 1 m in Case 2. In both, the BCVA was 0.00 logMAR in the RE.

In both cases, at slit lamp examination, the LE showed failed grafts with diffuse corneal stromal oedema, Descemet membrane folds, and epithelial bullae. Anterior chamber (AC) visualisation was limited, and a fundus examination was performed with B-scan ultrasonography, which showed a flat retina. RE examination was unremarkable.

To better assess the LE cornea, both AS-OCT (CASIA SS-2000, Tomey Corporation, Nagoya, Aichi, Japan) and IVCM (Heidelberg Engineering GmbH, Heidelberg, Germany) were performed. 

Using AS-OCT, Case 1 showed a central corneal thickness (CCT) of 697 µm and was positive for stromal ripples in the posterior corneal profile of the failed graft, while in Case 2, the CCT, excluding the DSAEK graft, was 1076 µm (1259 µm including the graft) [6].

IVCM showed fluid-filled epithelial bullae in the superficial epithelium and wing cells with hyper-reflective boundaries between cells with less epithelial bullae. In the stroma, there was loss of keratocytes in the anterior portion, while middle/deep stroma scanty keratocytes were noticed. It was not possible to assess the endothelium in view of the poor image quality due to the severity of corneal oedema.

Due to the chronic nature of the oedema and the high probability of graft failure, if a fourth endothelial graft was performed, the implantation of an EndoArt device was proposed for the affected eye. 

The procedure was performed as follows under local anaesthesia: The first step was to enhance surgical visibility by debriding the corneal epithelium using a hockey stick spatula, followed by a main temporal incision using 2.75 mm keratome and a superior side port using a 1.2 mm corneal knife. Peripheral iridectomy was already present, likely due to previous DSAEK grafts. Descemetorhexis with the same size as the PK graft in Case 1 and the removal of the DSAEK graft in Case 2 were performed under continuous irrigation with a balanced salt solution placed via a side port. Subsequently, the EndoArt implant was placed on the corneal surface to check its correct orientation, shown by the “F” mark. The delivery of the implant into the anterior chamber was performed using a spatula with a blunt tip with a single-hand “sliding and pushing” manoeuvre. This manoeuvre consists of placing the spatula on top of the implant placed on the corneal surface, pushing on the surface of the cornea, and then sliding it back towards the main incision and pushing it into the anterior chamber with the help of continuous irrigation placed under the implant during its delivery. The implant then spontaneously unfolds. After delivery, the irrigation cannula is removed from the side port, the correct orientation of the implant is checked, the AC is filled with 12% perfluoropropane (C_3_F_8_), and the implant aligned with the visual axis using a blunt hook. The delivery and centration of the EndoArt implant are reported in Video S1 (Supplementary Materials).

The main incision and side port were sutured, and one transfixing anchoring 10-0 suture through the host cornea and the implant was placed. If needed, refill of the AC with C_3_F_8_ was performed. At the end of the procedure, a soft bandage contact lens (BCL) was placed, and the patients were kept lying flat and looking upwards for 20 min in the operating theatre.

As post-operative treatment, patients were prescribed topical dexamethasone 0.1% and netilmicin 0.3% six times a day for 2 weeks, which then tapered to four times a day for the first month. The antibiotic treatment continued four times per day until the discontinuation of the BCL, and the topical steroid was tapered down. The BCL was replaced every 2 weeks, and the removal of the anchoring suture and BCL was planned at a 6-month follow-up time point.

Follow-ups were scheduled at days one, two, and three, and then weekly for 1 month and every two weeks thereafter. No EndoArt detachment requiring rebubbling or post-operative complication occurred [7].

At the last follow-up available (3-month), the BCVA value improved in Case 1 from 1.50 logMAR to 0.30 logMAR, while in Case 2, from CF to 1.70 logMAR. Additionally, in both cases, a subjective improvement in discomfort was reported.

**Figure 1:**
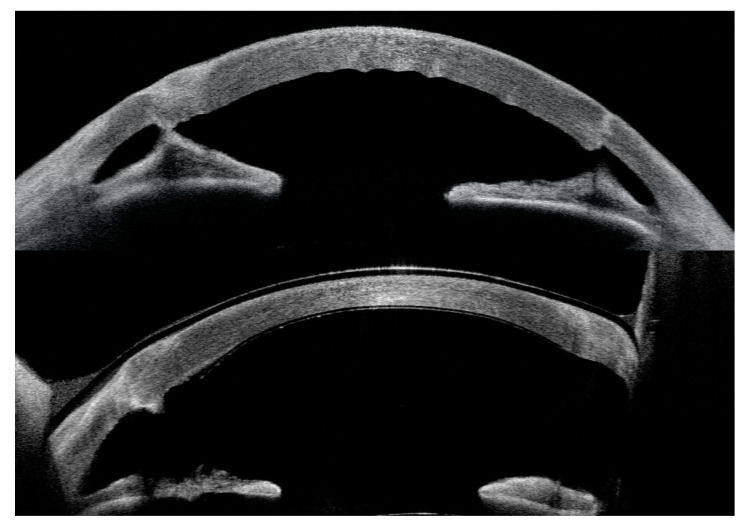
Case 1. AS-OCT before EndoArt implantation (on **top**) and at the 3-month follow-up (**bottom**). It is possible to see the resolution of stromal ripples, which were present at baseline; the resolution of oedema; and the reduction in the corneal thickness.

**Figure 2:**
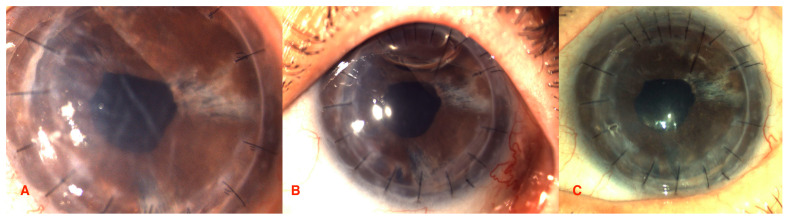
Case 1. Slit-lamp images before EndoArt implantation (**A**) at 3 weeks, (**B**) at 3 months, and (**C**) post-implantation.

**Figure 3:**
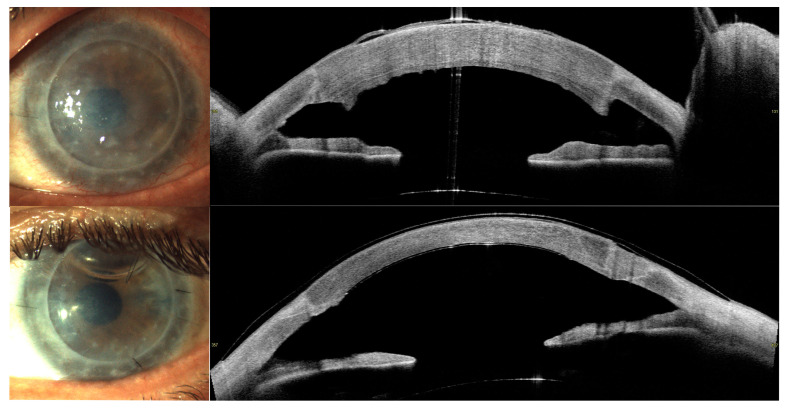
Case 2. Slit-lamp images and AS-OCT before EndoArt implantation (on **top**) and after implantation (**bottom**).

Upon examination, the resolution of epithelial bullae was noticed at the 1-week follow-up, with the progressive resolution of corneal oedema over 1 month, followed by overall stability (Figure 1, Figure 2 and Figure 3). CCT, excluding the implant, decreased by 28.7% in Case 1 from 697 μm (pre-operative) to 602, 509, 504, and 496 μm, respectively, at 1 week, 1 month, 2 months, and 3 months. In Case 2, the CCT was reduced by 34.3% from 1076 μm (pre-operative) to 745 μm at 3 months.

**Figure 4:**
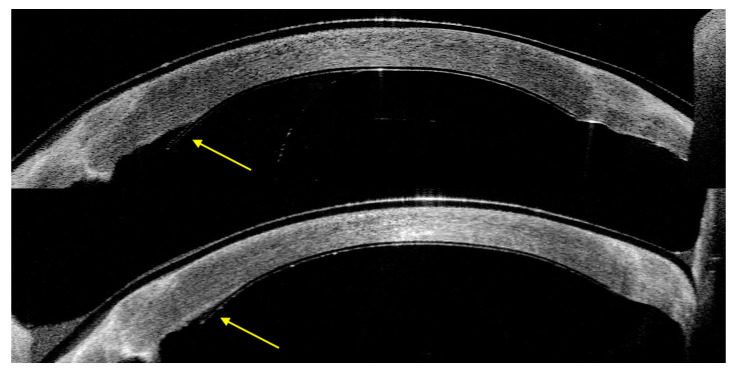
Case 1. Small detachment of EndoArt (yellow arrow), which became stable over time (day 2 on **top** versus 3 months at **bottom**).

In Case 1, AS-OCT showed a quick resolution of the posterior stromal ripples at day 2 post-operative. Additionally, a small peripheral inferior nasal detachment of the device, from 6 to 8 o’clock, at the level of PK graft–host junction was noticed at day one, which was stable during the follow-up (Figure 4).

**Figure 5:**
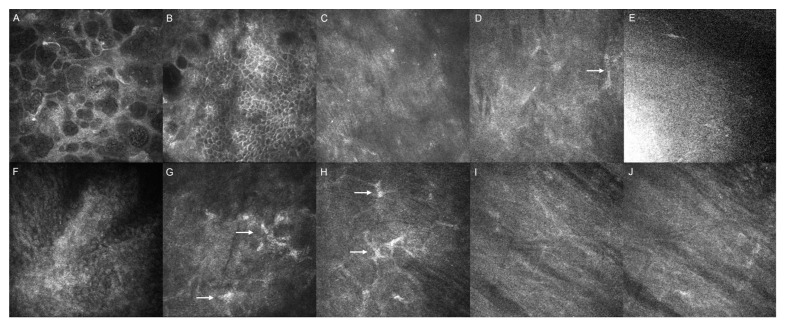
Case 1. In vivo confocal microscopy images pre-implantation and at 2 months post-EndoArt implantation. (**A**–**E**) Pre-op images. (**A**) superficial epithelium (depth 1 µm) showing fluid-filled epithelial bullae; (**B**) wing cells (depth 17 µm) displaying hyper-reflective boundary between cells with less epithelial bullae; (**C**) loss of keratocytes in superficial stroma (68 µm); (**D**) scanty keratocyte seen (arrow) in middle/deep stroma (depth 283 µm); and (**E**) poor quality image of endothelial layer (577 µm) due to severity of cornea oedema. (**F**–**J**) Post EndoArt implantation. (**F**) Superficial epithelium (depth 1 µm) showing disappearance of epithelial bullae and hyper-reflective region with surrounding epithelial cells; (**G**) early repopulation of hyper-reflective keratocytes (arrows) in superficial stroma (depth 86 µm); (**H**) similar repopulation of keratocytes (arrows) in deep stroma (298 µm); (**I**,**J**) imaging of EndoArt implant showing amorphous appearance with heterogenous reflectivity and absence of cellular structure (depths of 614 and 655 µm).

At the 2-month follow-up, in Case 1, IVCM was performed. The superficial epithelium showed the disappearance of epithelial bullae and a hyper-reflective region with surrounding epithelial cells. The early repopulation of hyper-reflective keratocytes was present in both superficial and deep stroma. The IVCM imaging of the EndoArt implant shows an amorphous appearance with heterogenous reflectivity and the absence of a cellular structure (Figure 5).

Retina OCT (Spectralis; Heidelberg Engineering, Heidelberg, Germany) was performed in both patients at the follow-up visits, which showed flat macula with no cystoid macular oedema.

### 3.2. Literature Review

A total of six articles met the research criteria [3,4,5,6,7,8], and of these, five reported functional outcomes of EndoArt [3,4,5,7,8]. Including the 2 cases presented in this manuscript, overall, a total of 23 cases of EndoArt are reported in the literature. A total of 82% of cases had previous history of at least one corneal transplant, while 39% had ≥3 transplants, and 78% had ocular comorbidities possibly affecting visual acuity (VA) outcome, with previous glaucoma surgery being the most common (83%). In the 23 cases, 46 corneal transplants were already performed before the EndoArt implant.

The follow-up ranged from 1 to 17 months (median 3 months). After EndoArt implantation, the mean reduction in the CCT was 29%, and the mean rebubbling rate was 47.8% (ranging from 0 to 100%). Seven cases (63.6%) required one rebubbling procedure, while four cases (36.4%) required more than one; 12% C_3_F_8_ was the preferred tamponade used (in 74% of cases), while 20% sulfur hexafluoride (SF_6_) was used in 26% of cases.

At least one transfixing 10-0 nylon anchoring suture was placed in 21 out of 23 cases (91.3%). It was not possible to perform an analysis of the rebubbling rate according to the type of tamponade or number of anchoring sutures placed due to a lack of detail in the reported cases [7].

VA improved in 69.6% of cases and remained unchanged in 13%, while it reduced in 17.4%. An improvement in pain symptoms was assessed in the cases in our study and in the study conducted by Fontana et al., with 100% of the cases experiencing pain relief after implantation [4].

Overall, the BCVA value improved from 1.61 ± 0.5 logMAR to 1.07 ± 0.59 logMAR (*p<* 0.001), while the CCT decreased from 771 ± 146 µm to 580 ± 134 µm (*p* < 0.001). Considering the subgroup analysis for VA, in cases of improvement, it improved from 1.68 ± 0.43 logMAR to 0.92 ± 0.51 logMAR (*p* < 0.001), while in cases of reduction, it reduced from 1.25 ± 0.69 logMAR to 1.45 ± 0.65 logMAR (*p* = 0.016). From the BCVA analysis, the two cases in the study by Auffarth et al. were excluded in view of a lack of VA reports at the last follow-up appointment at 17 months; however, the authors reported subjective improvements in VA [3]. VA reported in HM and CF were converted in logMAR [10].

Considering post-operative complications, cystoid macular oedema was the most common (three cases, 13%), followed by raised IOP (two cases, 8.6%). In only one case (4.3%), the removal of EndoArt was performed due to the persistency of corneal oedema after three device rebubbling attempts. Of note, one case (4.3%) experienced subepithelial corneal opacity, which was treated with phototherapeutic keratectomy, and no EndoArt implant removal was performed. No immune reaction or excessive fibrotic response was reported.

Full details of the included studies and outcomes, including the two case reports reported in this manuscript, are shown in Table 1 and Table 2.

## 4. Discussion

EK continues to gain popularity because of its advantages, including quick visual recovery, more predictable refractive results, and greater structural integrity compared to traditional PK [11]. Considering that the long-term prognosis of PK largely depends on the number of previous graft failures, EK surgery is preferred after failed PK [11]. Despite the benefits of DMEK for patients after PK, high rates of graft detachment have been reported, making it the most common reason for graft failure [2]. As a consequence, the management of chronic corneal oedema can be challenging, with increased risks for patients due to repeated surgical procedures, with an additional impact on economic burden and corneal tissue availability. Subsequently, novel devices such as EndoArt may represent a useful alternative in selected cases.

The outcomes of EndoArt are starting to be reported in the literature, although the data are currently limited to a total of 23 eyes (Table 1). Reduction of CCT after implantation ranged between 18 and 37% (mean reduction of 28%) with a maximum follow-up of 17 months. If we compare this to the reduction in the CCT over time in cases of DMEK and DMEK on PK, the values are, respectively, 18 and 30.2% at six months and 17.4 and 30.3% at one year [12,13]. We did not report the outcomes of DSAEK in view of the influence of lamellar donor tissue on the corneal thickness. Future comparative studies may be beneficial to determine the rate of CCT reduction, namely whether it is faster in cases of EndoArt or DMEK, as well as whether it is stable over time with a longer follow-up.

In the two patients in our study, we did not observe implant device detachment requiring rebubbling, but it has been reported in 11 out of 23 cases (47.8%), with a total of 21 rebubbling procedures (Table 1). The subgroup analysis (Table 3) excluded the 12 cases reported by Wiedemann et al. in view of a lack of information regarding at which follow-up time rebubbling was performed, but it nevertheless showed interesting findings. Seven out nine rebubbling procedures (77.8%) occurred between week 3 and month 3 after implantation, while just one occurred between month 3 and month 6 [3,4,5,8]. Just two cases were reported within the first 2 weeks [4,8]. Compared to EK, where graft detachment requiring rebubbling is an early post-operative complication more common in the first two weeks, after EndoArt, this may occur at a later time point, necessitating a strict follow-up, especially in the first 3 months [14].

Further research is needed to better investigate the risk factors and which type/extension of detachment needs rebubbling. Indeed, in our case, a small detachment was present in Case 1, and it was stable over time, with no alteration in the CCT; therefore, we did not perform rebubbling. Also, it should be considered that currently, EndoArt is only used in complex eyes with a history of multiple previous surgeries and/or the presence of a glaucoma drainage device (GDD), which could increase the risk of detachment.

Further research must investigate the role and number of transfixing, anchoring sutures and whether they are correlated with the rebubbling rate. Indeed, to date, there is no consensus on the number required, as it varies from zero to three, nor when they should be removed, and whether the risk of rebubbling is increased thereafter. The results regarding this aspect are still limited.

Generally, the sutures are reported to be removed at 3 months [4,5,7]. Considering the current literature, cases with a follow-up longer than 3 months are only reported by Fontana et al., Auffarth et al., and Abusayf et al., with a total of eight EndoArt cases [3,4,5]. Of these, the two cases reported by Auffarth et al. did not have sutures and required rebubbling at week 3 and month 1; Abusayf et al. (1 case) placed three sutures, which were removed between months 3 and 4, and no rebubbling was reported at any follow-up point, while Fontana et al. (5 cases) placed one suture, and only one rebubbling procedure was reported from month 3 to month 6.

Considering the functional outcomes, the results are promising, although they are limited by the presence of complex ocular comorbidities, which may have an impact on the final BCVA value, which also represents a possible confounding factor. Overall, the BCVA value improved from 1.61 ± 0.5 logMAR to 1.07 ± 0.59 logMAR, and interesting results were obtained in the sub-group analysis. A total of 70% of patients experienced a better BCVA value after implantation, with a significant improvement from 1.68 ± 0.43 logMAR to 0.92 ± 0.51 logMAR, while 17.4% reported a reduction in the BCVA value with a moderate worsening from 1.25 ± 0.69 logMAR to 1.45 ± 0.65 logMAR.

So far, EndoArt has only been used in eyes with a history of multiple surgeries, and VA outcomes cannot be compared with endothelial keratoplasty [12,15,16]. Further research on the use of EndoArt in cases of primary endothelial corneal decompensation not already managed with keratoplasty are needed.

A possible additional use for EndoArt could be for pain relief purposes in the presence of end-stage corneal decompensation in eyes with poor visual acuity potential in view of the reported resolution of pain symptoms in our study and in the study by Fontana et al. [4].

Limited complications occurred after implantation, with CMO being the most prevalent (13%), which is still in the upper range of CMO after DMEK, as it is between 4.8 and 13.8% [17,18,19,20,21]. In only one case, the implant was removed after three rebubbling attempts and the persistence of corneal oedema, and no cases of immune rejection of the device occurred, at least in the short term.

Moving from the outcomes to the surgical technique, apart from the number of transfixing sutures, which was already discussed, different delivery methods have been reported. The most common method is to use a spatula with a blunt tip to slide and push the implant in the anterior chamber [4,5,7]. “Push-in” and “pull-through” techniques have also been used with, respectively, an intraocular lens injector and intraocular forceps or glide, loading the EndoArt with the concave side facing upwards [3,5,8].

In cases of eyes with poor lens–iris diaphragm support, implant delivery may suffer from the same complication as DMEK in vitrectomised/aniridic/aphakic eyes, with the dislocation of the implant in the posterior chamber [15]. Indeed, this occurred in the case in the study by Abusayaf et al., which required pars-plana vitrectomy (PPV) following a re-implant of another EndoArt device at the same time of the dislocation of the first. The authors used a “pull-through” technique for the second device and, as reported, no dislocation or rebubbling was needed. The dislocated device was elevated from the posterior to anterior chamber at the time of PPV and removed through a corneal paracentesis [5].

Considering instead our results of corneal morphological changes in AS-OCT and IVCM before and after the implant, there are a few considerations. In AS-OCT, we observed a fast resolution of the posterior stromal ripples, which disappeared at day 2. These are irregularities in the posterior corneal profile that assumed the shape of a ripple and could be related to the composition of the stroma and a different ratio of dermatan to keratan sulphate [22]. Indeed, the ratio is higher in the anterior stroma rather than the posterior, and dermatan sulphate is less hydrophilic than keratan sulphate [23,24].

In our previous research, we highlighted that the presence of posterior stromal ripples is related to a higher risk of DMEK graft detachment. The resolution of the ripples after EndoArt may be related to the fast reduction in corneal oedema with the de-hydration of the keratan sulphate. We still cannot speculate whether post-operative persistent posterior stromal ripples are related to a higher risk of EndoArt detachment in view of the limited number of cases.

IVCM showed changes in both the epithelium and stroma. In the epithelial layer, there was a reduction in hyper-reflectivity and disappearance of the epithelial bullae as consequences of oedema resolution. In the stroma, the repopulation of keratocytes may be due to cell migration or the reduction in stromal swelling with a subsequent increase in their density. Additionally, after implantation, the presence of needle-shaped hyperreflective structures was noticed in the stroma and not before the implant likely due to poor visualisation in view of the oedema. These have been postulated to be crystalline or lipofuscin deposits, which tend to resolve over time [25,26]. Compared to IVCM after DMEK/DSAEK, in cases of EndoArt, there is a lack of hyper-reflective bands at the host–donor interface [25,26]. No endothelial cell migration over the device was noticed in IVCM.

## 5. Conclusions

In conclusion, our observations show that EndoArt can be a valid approach in cases of chronic oedema not only in the presence of failed DSAEK/DMEK, but also in failed PK. The device’s biocompatibility could overcome the high rejection rate of corneal transplantations and the shortage of donor corneas despite reports of high rebubbling rates. Further studies with a larger number of patients are needed to ascertain the role of EndoArt for this and further clinical indications.

## Figures and Tables

**Table 1:** Outcomes of EndoArt.

Study	Follow-Up (Months)	Number of Cases	Tamponade Used	Number of Transfixing Anchoring Sutures Placed per Eyes	Rebubbling Rate	Number of Rebubbling Procedure	Pre-Operative Central Corneal Thickness (µm)	Final Central Corneal Thickness (µm)	Rate of Reduction in Central Corneal Thickness	Visual Acuity Improved	Pre-Operative Visual Acuity (logMAR)	Final Visual Acuity (logMAR)
Auffarth et al. [3]	17	2	20% SF_6_	0	100%	2	745 ± 22	491 ± 49	34%	n/a	Case 1: HM Case 2: 1.1	n/a
Fontana et al. [4]	6	5	12% C_3_F_8_	1	80%	6	805 ± 135	588 ± 60	27%	Yes	1.26 ± 0.25	0.74 ± 0.44
Abusayf et al. [5]	12	1	12% C_3_F_8_	3	0%	0	911	691	24%	No	CF	0.7
Kobayashi et al. [8]	3	1	20% SF_6_	1	100%	1	845	530	37%	Yes	HM	2
Wiedemann et al. [7]	3	12	12% C_3_F_8_ (9 cases) 20% SF_6_ (3 cases)	1–3	33%	12	719 ± 145	591 ± 190	18%	Yes	1.6 ± 0.5	1.1 ± 0.6
Our report	3	2	12% C_3_F_8_	1	0%	0	887 ± 268	621 ± 176	30%	Yes	Case 1: 1.50 Case 2: CF	Case 1: 0.30 Case 2: 1.70

CF: counting fingers; HM: hand movement; n/a: not available.

**Table 2:** The characteristics of the included studies.

Study	Year	Type of Study	Indication for EndoArt	Presence of Ocular Comorbidities Which May Affect Visual Acuity Outcomes	Post-EndoArt Implant Complications
Auffarth et al. [3]	2021	Case report	Failed DMEK	Case 1: previous endophthalmitis	No
Fontana et al. [4]	2023	Retrospective Case Series	Case 1: Failed DSAEK (2×) Case 2: Failed DSEK (2×) Case 3: Failed DSEK (3×) Case 4: Failed DMEK (2×) Case 5: Failed DSEK (2×)	Case 2: chronic post-operative CMO Case 3: previous glaucoma surgery (trabeculectomy) Case 4: previous glaucoma surgery (trabeculectomy), post-operative CMO and chronic raised IOP	CMO: 2 cases Chronic raised IOP: 1 case
Abusayf et al. [5]	2023	Case report	PBK	Juvenile open-angle glaucoma, traumatic aphakic glaucoma, pars-plana vitrectomy, multiple glaucoma surgeries	No
Kobayashi et al. [8]	2024	Case report	Failed DMEK	Epiretinal membrane	No
Wiedemann et al. [7]	2024	Retrospective case series	Presence of GDD (PreserFlo, Paul-tube, Baerveldt, or Ahmed implants) with endothelial decompensation or single/multiple DMEK failure Total of 26 DMEK were already performed in 12 cases included	History of glaucoma	Raised IOP: 1 case (treated with topical medications) CMO: 1 case Subepithelial corneal opacity: 1 case (treated with PTK) Removal of EndoArt implant: 1 case (persistency of corneal oedema following 3 rebubbling procedures)
Our report	2024	Case series	Case 1: Failed DSAEK (2×) and failed PK Case 2: Failed PK (3×) and failed DSAEK (1×)	No	No

CMO: cystoid macular oedema; DMEK: Descemet membrane endothelial keratoplasty; DSAEK: Descemet stripping automated endothelial keratoplasty; DSEK: Descemet stripping endothelial keratoplasty; GDD: glaucoma drain device; IOP: intraocular pressure; PBK: pseudophakic bullous keratopathy; PTK: phototherapeutic keratectomy.

**Table 3:** Subgroup analysis of rebubbling procedure performed.

	Fontana et al. (Case 1) [4]	Fontana et al. (Case 2) [4]	Fontana et al. (Case 3) [4]	Fontana et al. (Case 4) [4]	Fontana et al. (Case 5) [4]	Auffarth et al. (Case 1) [3]	Auffarth et al. (Case 2) [3]	Abusayf et al. [5]	Kobayashi et al. [8]
Day 1									X
Week 1									
Week 2	X								
Week 3				X					
Month 1			X				X		
Month 2		X							
Month 2.5	X								
Month 3				X					
Month 6						X			

X: rebubbling performed. Total: 9.

## Data Availability

The datasets are available upon reasonable request.

## Supplementary Materials

The following supporting information can be downloaded at: https://www.mdpi.com/article/10.3390/jcm13216520/s1, Video S1: Delivery and Centration of EndoArt.
